# An evidence‐based approach to the routine use of optical coherence tomography

**DOI:** 10.1111/cxo.12847

**Published:** 2018-12-17

**Authors:** Angelica Ly, Jack Phu, Paula Katalinic, Michael Kalloniatis

**Affiliations:** ^1^ Centre for Eye Health, The University of New South Wales Sydney New South Wales Australia; ^2^ Faculty of Science, School of Optometry and Vision Science, The University of New South Wales Sydney New South Wales Australia

**Keywords:** age‐related macular degeneration, diabetic retinopathy, glaucoma, imaging, screening

## Abstract

Optical coherence tomography is an imaging technology that has revolutionised the detection, assessment and management of ocular disease. It is now a mainstream technology in clinical practice and is performed by non‐specialised personnel in some settings. This article provides a clinical perspective on the implications of that movement and describes best practice using multimodal imaging and an evidence‐based approach. Practical, illustrative guides on the interpretation of optical coherence tomography are provided for three major diseases of the ocular fundus, in which optical coherence tomography is often crucial to management: age‐related macular degeneration, diabetic retinopathy and glaucoma. Topics discussed include: cross‐sectional and longitudinal signs in ocular disease, so‐called ‘red‐green’ disease whereby clinicians rely on machine/statistical comparisons for diagnosis in managing treatment‐naïve patients, and the utility of optical coherence tomography angiography and machine learning.

In a short 27 years, optical coherence tomography (OCT) has become an indispensable, modern‐day tool for the comprehensive evaluation of ocular disease and diseases of the visual pathway. It is a diagnostic imaging technology that provides a swift, high‐resolution, and non‐invasive cross‐sectional view of microscopic, anterior and posterior ocular structures.^1^ The technology has progressed from time domain to spectral domain, and swept‐source models are also now available. The shift from time domain to spectral domain OCT saw the removal of the need for mechanical movements of the reference arm, thus achieving faster, higher‐quality scans with fewer artefacts and greater resolution. OCT angiography (OCT‐A) represents the latest in the chain available in clinical practice, with the capacity to provide a unique, non‐invasive view of retinal and choroidal vasculature, and has demonstrated a further potential to alter clinical practice patterns.^2^, ^3^

Several OCT adjuncts are available, including: options for fundus viewing (with some instruments providing a wide‐field option), enhanced depth imaging of the choroid and sclera, averaging to improve the signal‐to‐noise ratio, live tracking and image registration. Software analysis algorithms are also manifold, ranging from thickness or volumetric analyses (total or layer‐by‐layer), normative comparison, asymmetry analyses, automated landmark detection (of structures such as the fovea centre, optic cup and disc), visualisation options (colour rendering, *en face*, movie or three‐dimensional reconstructions), and integrity analyses specific to a single layer or band, for example, the Cirrus OCT ‘advanced retinal pigment epithelium (RPE) analysis’. In Australia, the ability to use and interpret OCT now forms an entry‐level competency criteria into the optometric profession.^4^

## We can, but should we? The use of routine OCT for disease screening

OCT uses low‐coherence interferometry to generate a reflectivity profile in the axial direction, known as an A‐scan.^1^ Several adjacent A‐scans are compiled to produce a structural OCT B‐scan or histology‐like, cross‐sectional view of ocular topography. Finally, a volume of B‐scans may be assembled to produce a C‐scan, *en face* or front on view of the same tissue. OCT‐A images are formed using the decorrelation signal (amplitude and phase variables) of OCT B‐scans acquired in rapid succession and provide a non‐invasive ‘map’ of intrinsic ocular blood flow.^5^ Both *en face* OCT and OCT‐A images may also be tweaked to provide a discriminate, ‘dissected’ view of deeper retinal tissues with relatively little interference from inner structures. OCT may be generally applied for *in vivo* imaging of the cornea, anterior chamber, vitreous, retina, optic nerve head, macula and/or choroid, for a range of purposes, including: (1) the earlier detection of eye disease; (2) more accurate differential diagnosis of ocular disease, including more precise disease staging; or (3) change assessment over time.

The primary caveat regarding the use of OCT for any of these applications is that despite its resemblance, structural OCT B‐scans are not the same as histology. The resultant image is a representation of the optical rather than the histological staining properties of tissue structure and is bound by that same logic. For example, opaque structures will cause posterior shadowing, such as in the case of a melanotic choroidal naevus.^6^ Similarly, an irregular highly reflective surface such as drusen in the RPE/Bruch's membrane complex will backscatter and alter the visibility of Henle's fibre layer.^7^ Historically, there has also been considerable confusion regarding the origin of the layers and the descriptors for the various layers, bands and zones.^8^ For instance, the now dubbed ‘inner segment ellipsoid zone’ has been formerly described as the inner segment outer segment junction or the photoreceptor integrity line.^8^, ^9^ Clinicians should also be wary of any quantitative data generated from these qualitative images. Each measurement may be compared against a normative database and using the manufacturers’ conventions, values colour‐coded green are interpreted as falling within normal limits. Red indicates a value outside of normal limits and as such could be described as abnormal or a ‘fail’ result. However, to use these tools effectively, clinicians should carefully consider the likelihood of false positives (red disease) or false negatives (green disease) in the context of the clinical view of the optic disc, macular and posterior pole, and take an active interest in scrutinising the raw data whenever available. Although time‐consuming, this step is key to avoiding errors in diagnosis and thus management. More recently, OCT‐A has been likened to fluorescein angiography. However, a key difference is that OCT‐A, although capable of visualising choroidal neovascularisation membranes, does not capture leakage.^10^, ^11^, ^12^

The purpose of this review is to provide a practical guide for the application of OCT to ocular disease assessment in treatment‐naïve patients, that is, patients most likely to be seen in a primary care setting who have not received any treatment for their chronic eye disease. Because the leading causes of blindness in Australia are age‐related macular degeneration (AMD), diabetic retinopathy (DR) and glaucoma,^13^ the application of OCT to these diseases forms the focus of this manuscript. Through illustrative cases of patients seen at the Centre for Eye Health,^14^, ^15^ we describe both the advantages and limitations of its use. We also emphasise the importance of an evidence‐based, multimodal imaging approach to eye care. Finally, we discuss the role of OCT as a diagnostic versus a screening test. Research‐related variations on OCT, not yet widely available in clinical practice, including polarisation‐sensitive OCT, projection‐resolved OCT‐A or multi‐directional OCT, will not be discussed.

All case images herein were captured under a standardised testing protocol (varying by the eye condition in question) combining: monoscopic and stereoscopic fundus photography (Kowa WX 3D non‐mydriatic retinal camera, Kowa, Tokyo, Japan), Optomap ultra‐widefield and fundus autofluorescence imaging (Optomap Panoramic 200Tx, Optos, Dunfermline, Scotland, UK), OCT (Cirrus HD‐OCT, Carl Zeiss Meditec, Dublin, California, USA and/or Spectralis HRA2 + OCT, Heidelberg Engineering, Heidelberg, Germany) with scan spacing and density at the discretion of the examining clinician, and standard automated perimetry (Humphrey visual field analyzer, Carl Zeiss Meditec). Patient written consent was obtained in accordance with the Declaration of Helsinki and approved by a Biomedical Human Research Ethics Advisory Panel of the University of New South Wales, Australia.

## Age‐related macular degeneration

### Early to intermediate AMD

**Figure 1:**
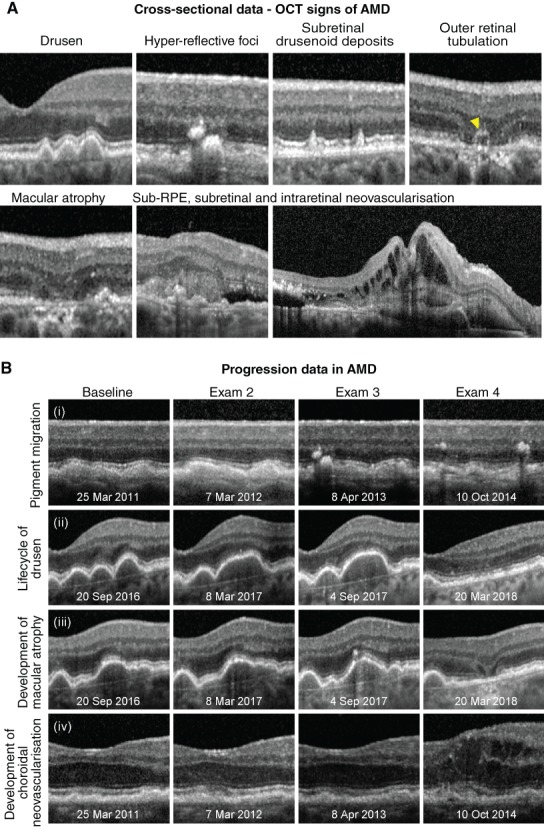
A: Optical coherence tomography (OCT) signs of age‐related macular degeneration (AMD). Drusen or elevations of the retinal pigment epithelium (RPE)/Bruch's membrane complex, hyper‐reflective foci in the outer retina and subretinal drusenoid deposits are key risk factors for progression, typical of intermediate AMD, and may be identified using OCT data acquired from a single patient attendance. In contrast, atrophy (in this instance, complete thinning of the outer nuclear layer and dropout of the ellipsoid zone and RPE with and without outer retinal tubulation; yellow arrowhead), incommensurate sub‐ or intra‐retinal fluid (appearing as optically empty spaces) and subretinal hyper‐reflective material represent OCT signs of advanced AMD. B: Four eyes illustrating the clinical application of OCT for change analysis in AMD. Each row shows a common sequence of events that precede progression to advanced disease: (i) the emergence of hyper‐reflective foci overlying drusen followed by pigment migration, (ii) confluence of drusen over time followed by regression, (iii) emergence of hyper‐reflective foci followed by the development of nascent geographic atrophy, (iv) shallow drusenoid pigment epithelial detachment with eventual development of drusen substructures and intra‐retinal fluid; examination dates appear at the bottom of each image.

A current clinical classification scale^16^ recommends subdividing AMD into three stages: early, intermediate and late. Large drusen (> 125 μm in diameter) and pigmentary abnormalities define the intermediate stage, while macular atrophy and/or neovascularisation marks the conversion to late‐stage disease (Figure 1A). Using OCT, drusen in intermediate AMD typically appear as nodular elevations of the RPE/Bruch's membrane complex with medium internal reflectivity.^17^ Pigmentary abnormalities, on the other hand, usually coincide with hyper‐reflective foci in the outer retina and variations in RPE thickness.^18^, ^19^ The overlying outer nuclear layer and other photoreceptor layers (external limiting membrane and the ellipsoid zone) often exhibit associated attenuation and thinning. Scrutiny of the photoreceptor layers is important and may be considered in conjunction with drusen height, ultrastructure, area and volume as predictive ‘biomarkers’ of disease progression.^20^, ^21^, ^22^ For example, subretinal drusenoid deposits confer a 2–6‐fold higher risk of progression to late AMD.^23^, ^24^, ^25^ Choroidal thickness is also garnering increasing interest and may hold prognostic value for identifying patients who have or are at risk of developing AMD‐related macular atrophy (particularly if the subfoveal choroidal thickness is ≤ 124 μm).^26^ Using OCT‐A, non‐neovascular AMD may present with large areas of signal void and reduced flow signal in the choriocapillaris (due to flow velocities below the decorrelation threshold and/or decreased choriocapillaris vessel calibre and density).^27^, ^28^

### AMD‐related macular atrophy

AMD‐related macular atrophy (including geographic atrophy), on structural OCT, should span at least 250 μm and appears as thinning or dropout of the outer nuclear layer, ellipsoid zone and/or RPE with posterior hyper‐transmission.^29^ Incomplete and complete variations, with variable degrees of RPE loss and irregularity, have also been recently described.^29^ Incomplete denotes discontinuity in the findings, while complete describes more absolute homogeneity. In the junctional zones surrounding atrophy, OCT and OCT‐A provide an additional means of predicting the rate of disease progression by showing outer retinal and RPE alterations beyond the area of visible atrophy, much like fundus autofluorescence.^27^, ^30^

### Neovascular AMD

In neovascular AMD, OCT has been heralded as indispensable with a sensitivity of at least 90 per cent and high inter‐ and intra‐observer agreement.^31^, ^32^ In suspecting neovascular AMD, the clinician should carefully evaluate the subretinal and sub‐RPE space for fluid or incommensurable hyper‐reflective material (blood or fibrovascular tissue). Any intra‐retinal fluid or cystic spaces are also untoward and a screening strategy based on a minimum inter‐scan distance of 240 μm using spectral domain or swept source OCT (rather than time domain) is ideal for the detection of treatment‐relevant exudative signs.^33^, ^34^, ^35^ Small pockets of subretinal fluid inbetween adjacent drusen not exceeding their peaks occurs in 11 per cent of eyes with intermediate AMD^36^ and should be interpreted as a subclinical variation of choroidal neovascularisation mandating close surveillance or referral. The progression of intermediate AMD to advanced disease over time may also be facilitated using OCT by combining a working background knowledge of these key signs and their significance, with careful simultaneous comparison of sequential B‐scans (Figure 1B).

Advancement of neovascular AMD is typified by widespread morphological changes in foveal contour, subretinal fluid, fibrosis, pigment epithelial detachment and/or a diffuse increase in retinal thickness > 250 μm.^37^ Outer retinal (nuclear) tubulation describes any round or ovoid hypo‐reflective space with hyper‐reflective surrounds in the outer nuclear layer, and should be readily distinguished from intra‐retinal cysts.^38^ Outer retinal tubulation is common although not specific to advanced AMD and is often recalcitrant to anti‐vascular endothelial growth factor therapy, while intra‐retinal cysts indicate active disease requiring treatment.^39^

### The emergence of OCT‐A

**Figure 2:**
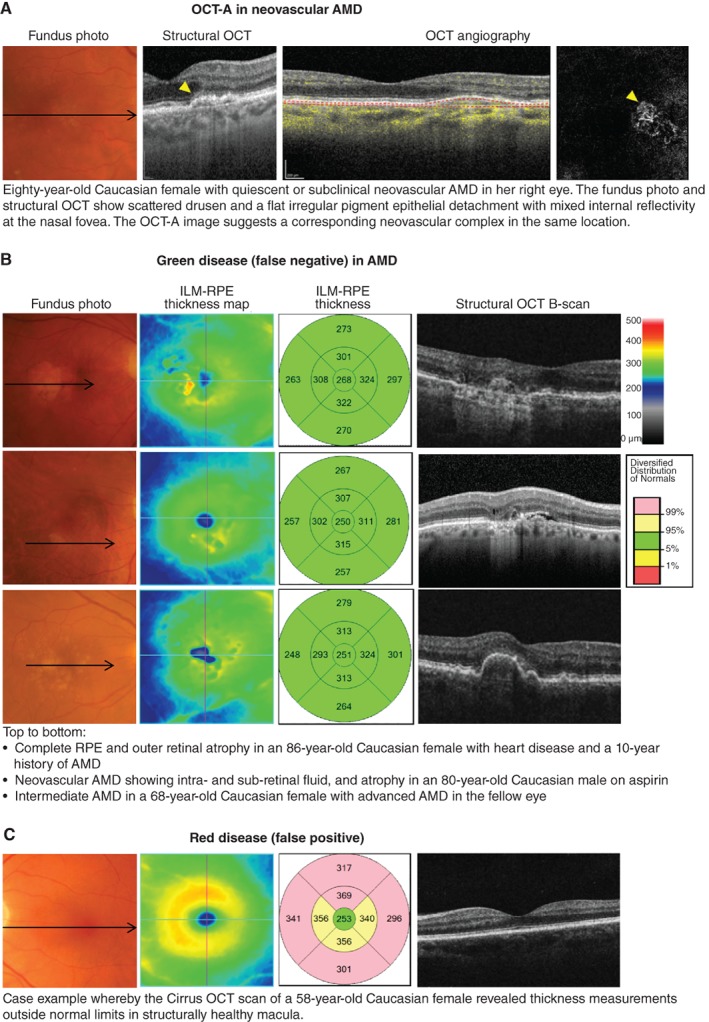
A: Case images illustrating the emerging application of optical coherence tomography angiography (OCT‐A) in age‐related macular degeneration (AMD). B: Examples of green disease (false negatives) that is cases where the internal limiting membrane (ILM)‐retinal pigment epithelium (RPE) thickness values fall within normal range despite the presence of significant AMD signs, reflecting that the injudicious reliance on macular thickness measurements is not recommended. C: Case example of OCT‐rendered red disease (false positives) at the macula.

The gold standard to date for the diagnosis of neovascular AMD is fluorescein angiography; however, a limitation of both fluorescein angiography and structural OCT is that they enable only indirect visualisation of choroidal neovascularisation‐related signs and do not allow visualisation of the membrane itself.^10^, ^11^, ^12^ Dye studies are also invasive and carry a rare, albeit not negligible, risk of anaphylaxis. Using OCT‐A, choroidal neovascularisation can be visualised non‐invasively typically in the normally ‘avascular’ space between the outer border of the outer plexiform layer and Bruch's membrane. The evolution of OCT‐A has re‐invigorated the conversation regarding the sub‐group of conditions, known as quiescent, non‐exudative neovascular, or ‘subclinical’ choroidal neovascularisation (Figure 2A).^40^, ^41^, ^42^ These cases describe eyes with demonstrable choroidal neovascularisation using fluorescein angiography, indocyanine green angiography or OCT‐A without exudation that may be safely followed without treatment. The inverse scenario whereby choroidal neovascularisation may not be visible on OCT‐A despite the presence of exudative signs is common and may often be ascribed to projection artefacts, media opacities or a masquerading condition of choroidal neovascularisation, including other common causes of macular oedema, such as central serous chorioretinopathy, myopic maculopathy (haemorrhage in the absence of choroidal neovascularisation) or DR.^11^

### The connection between clinical management and OCT signs of AMD

Current clinical guidelines recommend monitoring intermediate AMD and geographic atrophy every 6–18 months.^43^, ^44^ Modifiable risk factors (such as smoking) should be addressed and Age‐Related Eye Disease Study 2 nutritional supplements and Amsler grid self‐monitoring should also be recommended. The defining features of at least large drusen and pigmentary abnormalities may be easily identified using OCT; however, both signs within themselves are non‐specific and occur in other macular diseases. Subretinal drusenoid deposits on OCT and/or macular atrophy may alert the clinician to a higher‐risk phenotype of AMD but also occur, albeit rarely, in the absence of AMD.^45^ Sub‐retinal, intra‐retinal, and sub‐RPE fluid as occurs in neovascular AMD should be managed urgently and referred to a specialist within two weeks.^44^ However, as with the other signs, sub‐retinal and sub‐RPE fluid can also occur in other self‐resolving presentations such as acute central serous chorioretinopathy. In contrast, once the diagnosis of AMD is established, OCT provides an invaluable adjunct and supplement to the core testing, particularly for early detection and monitoring. Thus, a multimodal approach to ocular assessment in AMD, and mimicking disorders, is recommended.

### Limitations of OCT imaging in AMD

The signs of AMD, especially neovascular AMD, are commonly associated with OCT segmentation or centration errors.^46^ Both invalidate the automated thickness or volume measurements; the former does so by mis‐identifying either the inner or outer boundary while the latter implies inaccurate identification of the fovea and thus a misplacement of the calculation grid. These errors are likely to be more significant in the presence of low signal strength, advanced disease or increased retinal thickness.^47^, ^48^ Thickness values between instruments should also not be compared, not least due to limitations in agreement that may relate to variations in the segmentation boundaries used and the segmentation error rate.^49^ In the absence of errors, change in macular thickness over time may inform management decisions. Specific to AMD, OCT software algorithms for the automated measurement of drusen load in intermediate disease (area and volume) and geographic atrophy in late disease (area and proximity to the foveal centre) may also be clinically meaningful, particularly for disease monitoring;^50^, ^51^, ^52^ however, they are prone to the same decentration and segmentation errors.

**Figure 3:**
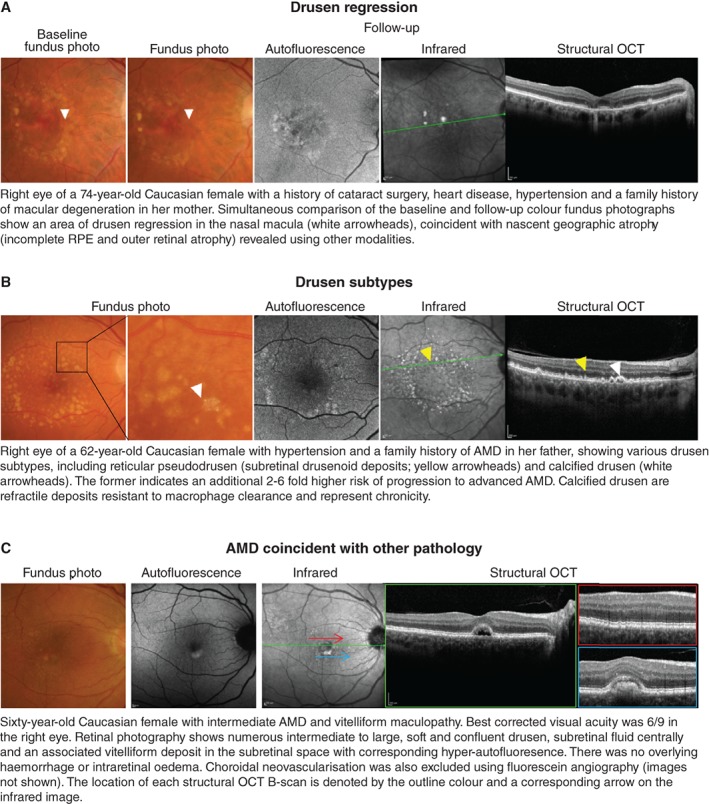
A: Drusen regression and B: drusen subtypes, especially reticular pseudodrusen or calcified drusen, represent significant risk factors for progression in age‐related macular degeneration (AMD) and are better followed using *en face* imaging methods and a multimodal imaging approach. C: Mimicking disorders and mixed presentations of disease are also better identified using an evidence‐based, multimodal imaging approach.

The signs of late AMD are often subtle and ill‐defined. To date, OCT‐A for the detection of choroidal neovascularisation has garnered interest but remains heavily user‐dependent, with sensitivities ranging between 50–85 per cent.^12^ Early detection using OCT still requires careful, systematic scrutiny of multiple layers. Injudicious reliance on global parameters, such as macular thickness values (Figure 2B, C), is not recommended, although other *en face* scans may be useful in providing a holistic view of the area and volume of involved regions. Significant signs, such as drusen regression,^53^ drusen subtypes^54^ or nascent geographic atrophy^55^, ^56^ may also be better identified using other methods (Figure 3A, B). Finally, the accurate interpretation and differentiation of AMD from other mimicking disorders often requires scrupulous inspection by appropriately trained staff with a thorough understanding of the pathophysiology, epidemiology and clinical characteristics of each condition (Figure 3C).

### Key points

- OCT may be usefully applied to identify treatment‐naïve cases at high risk of progression to late AMD.
- The OCT findings of cases suspected of neovascular AMD should be carefully examined for any sub‐retinal, sub‐RPE or intra‐retinal fluid using a minimum inter‐scan distance of 240 μm. OCT‐A is a useful adjunct for the detection of choroidal neovascularisation; however, sensitivity ranges between 50 and 85 per cent.
- *Limitations*: The signs of AMD are commonly associated with OCT segmentation or centration errors. Certain significant signs, including drusen regression, drusen subtypes or nascent geographic atrophy, are better identified using a multimodal imaging approach than OCT alone.

## Diabetic retinopathy (DR)

**Figure 4:**
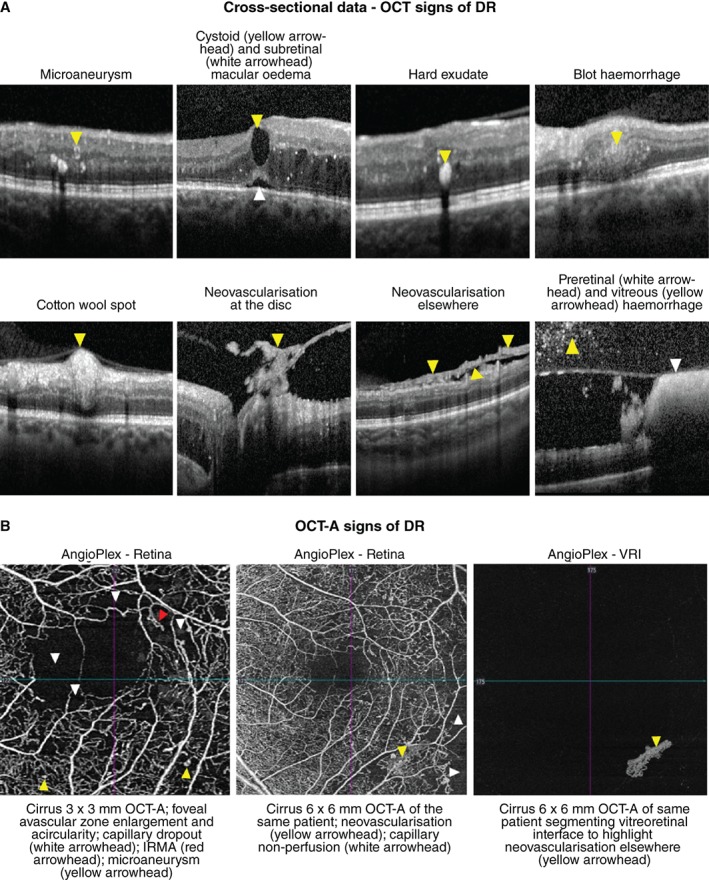
A: Key signs of diabetic retinopathy (DR) as they appear using optical coherence tomography (OCT) data attained at a single visit. Dilated fundus examination forms the basis of grading DR but is being increasingly supplemented by OCT to assist in differential diagnosis. B: OCT‐A (angiography) signs of DR. As pictured, microaneurysms may take on a range of shapes varying from nodular to earlobe‐like.

Clinically, DR is subdivided into non‐proliferative, proliferative, and diabetic macular oedema (DMO) which may occur at either the non‐proliferative or proliferative stage. The non‐proliferative sub‐type is characterised by specific ophthalmoscopic signs, including microaneurysms, dot or blot intra‐retinal haemorrhages, intra‐retinal microvascular abnormalities (IRMA), venous calibre abnormalities, cotton wool spots, and hard exudates (intra‐retinal lipid).^57^ Proliferative diabetic retinopathy is characterised by neovascularisation on the optic disc or elsewhere with or without pre‐retinal or vitreous haemorrhage.^58^ The number and distribution of lesions form the basis for current clinical classification systems.^57^, ^58^ While dilated fundus examination is the recommended method of grading DR using classifications such as the International Clinical Diabetic Retinopathy and Diabetic Macular Edema Disease Severity Scales,^58^ these techniques are increasingly being supplemented by advanced *en face* and cross‐sectional imaging techniques such as widefield scanning laser ophthalmoscopy and OCT (Figure 4A). More recently, OCT‐A has demonstrated a number of potential roles in DR diagnosis and management, such as identifying areas of retinal non‐perfusion often invisible with funduscopy and identifying potential biomarkers for disease severity (Figure 4B).

Microaneurysms are the earliest clinical sign of DR and may be undetectable using OCT, particularly if low‐density or low‐resolution volume scans are acquired. If visible, they typically appear on OCT as small round or oval lesions, usually within the inner nuclear layer, fully or partially capsulated in 56 per cent of cases.^59^ The majority of microaneurysms have moderate to highly hyper‐reflective lumen (81.4 per cent of cases)^60^ and are often located adjacent to intra‐retinal cystoid spaces. The hypo‐reflective variant occurs less commonly, in 18.6 per cent of cases. Microaneurysms are less visible with OCT‐A as compared to fluorescein angiography; as few as 62 per cent of microaneurysms detected with fluorescein angiography manifest within OCT‐A images, likely due to a lower rate of blood flow or absence of flow within the lesion.^59^, ^60^, ^61^ Within OCT‐A images, microaneurysms take on a range of shapes including nodular (65 per cent of cases), comma‐shaped, coil‐shaped, semilunar, crescent and earlobe‐like^62^ and the majority of microaneurysms are located within the deep capillary plexus.^60^, ^61^, ^62^

Clinically, microaneurysms are difficult to distinguish from intra‐retinal dot/blot haemorrhages, although OCT imaging shows the latter is typically located deeper in the retina and has a more amorphous shape.^59^ Hard exudates are intra‐retinal protein and/or lipid deposits which tend to appear as numerous hyper‐reflective deposits in the outer retina using OCT, often associated with intra‐retinal oedema. OCT imaging can also identify small hyper‐reflective foci that cannot be visualised with fundus biomicroscopy but show similar hyper‐reflectivity to hard exudates; these may represent precursors of hard exudates.^63^

### OCT for early detection of diabetic retinopathy and diabetic macular oedema

**Figure 5:**
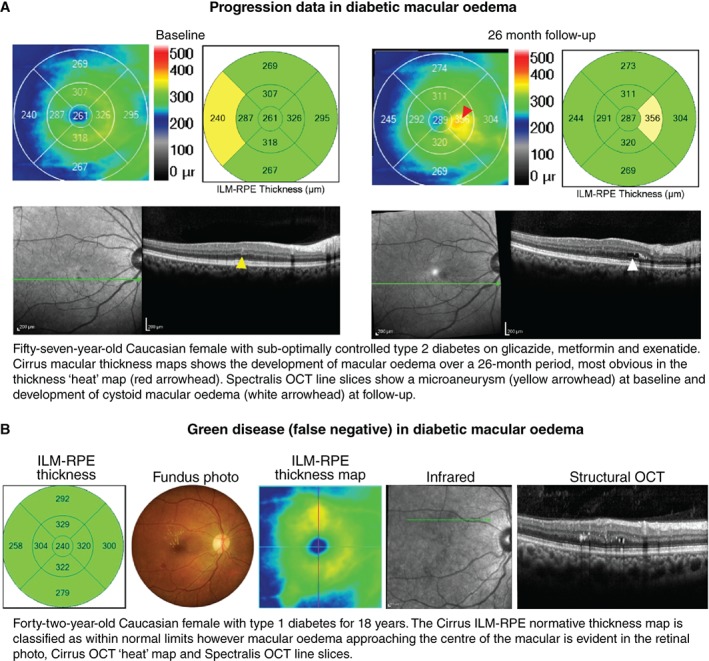
A: Case images illustrating the application of optical coherence tomography (OCT) for change analysis in diabetic retinopathy (DR). Note that the early detection of subtle cases such as these may be best appreciated using the retinal thickness maps. B: Green disease whereby other imaging modalities reveal consistent evidence of diabetic macular oedema although the normative analysis (left) using Cirrus OCT classifies all thickness values as within normal limits. Abbreviations: as with Figures 1, 2, 3, 4.

Diabetic macular oedema, defined as thickening of the macula due to accumulation of fluid resulting from a breakdown of the inner blood‐retinal barrier, is the most common vision‐threatening complication of DR. It can present in a focal, diffuse or cystic pattern of intra‐retinal oedema which may co‐exist with subretinal oedema.^64^ OCT can usefully show hypo‐reflective spaces within the inner and/or outer retinal layers or sub‐retinally when sufficient density scans are used. Alternatively, a diffuse or focal increase in retinal thickness may occur, better appreciated in the retinal thickness maps (Figure 5) or macular change analyses. With OCT‐A, pockets of intra‐retinal fluid may lead to decreased signal intensity or an apparent complete absence of flow.^65^

Early detection is vital to the prevention of vision loss and due to its high sensitivity OCT, as an adjunct to fundus examination for DR, is now widely regarded as indispensable for the detection of DMO and monitoring response to treatment. The International Council of Ophthalmology Guidelines for Diabetic Eye Care, updated in 2017, emphasise that the definition and classification of DMO should take into account OCT findings, if available.^66^ However, the high costs and training required preclude its use outside of high‐resource settings. There are also a number of proposed OCT‐based classification systems for DMO, not yet widely adopted, which in turn evaluate various parameters including retinal thickness and/or volume, presence/absence of hard exudates, location, morphology, vitreomacular interface changes and microstructural alterations.^63^, ^64^, ^67^, ^68^, ^69^

The term subclinical DMO was first described using time domain OCT (Stratus, Zeiss) by the Diabetic Retinopathy Clinical Research Network (DRCR.net) as central retinal thickness beyond 225 μm (not greater than 299 μm) in the absence of centre‐involving macular oedema detectable using slitlamp fundus examination.^70^ Of importance, 38 per cent of patients with these subclinical findings progressed to DMO or required treatment by two years. Subclinical DMO was later redefined by Diabetic Retinopathy Clinical Research Network using spectral domain OCT (Cirrus, Zeiss) as retinal thickness > 260 μm and < 290 μm in women and > 275 μm and < 305 μm in men.^71^ Directly comparing measurements from different OCT imaging technologies is problematic, due to differences in scan resolution, speed of acquisition and algorithms for locating the boundary of the outer retina, bringing into question the clinical usefulness of a numerical definition of DMO or subclinical macular oedema for eye care professionals in clinical practice.^72^ Nevertheless, increased central retinal thickness in the absence of other structural abnormalities may provide a biomarker for future progression to DMO.

Structural OCT has limited value in evaluating signs of severe non‐proliferative DR such as venous calibre abnormalities and intra‐retinal microvascular abnormalities; however, OCT may assist in the differential diagnosis of cotton wool spots, seen as a hyper‐reflective thickening of the retinal nerve fibre layer, as well as in confirming the presence of neovascularisation and determining the location of vitreous and pre‐retinal haemorrhage.^73^, ^74^ Using OCT, neovascularisation may appear as pre‐retinal hyper‐reflective material on the retinal surface or proliferating along the posterior vitreous interface. Vitreous haemorrhage can be seen as hyper‐reflective dots within the vitreous cavity and pre‐retinal (or sub‐hyaloid) haemorrhage may present as an area of hyper‐reflectivity trapped between the internal limiting membrane of the retina and the posterior vitreous interface.^74^

### The emerging role and limitations of OCT in DR

OCT‐A has shown promise as a non‐invasive method of ascertaining the morphology of intraretinal microvascular abnormalities and neovascularisation,^61^, ^75^ but outside the posterior pole the technique is limited by field of view and difficulty in off‐axis image acquisition. Akiyama et al.^76^ used OCT and OCT‐A to demonstrate that persistent vitreous attachment at the optic disc (that is absence of posterior vitreous detachment) is essential to the growth of neovascularisation at the optic disc, with neovascularisation proliferating along both sides of the posterior vitreous interface, and that neovascularisation arises from the neuroretinal rim or outside the optic disc margin rather than from the optic cup. Similarly, Vaz‐Pereira et al.^74^ found that 79 per cent of neovascularisation of the retina involved proliferation along the outer surface of the posterior vitreous interface, arising from areas of ischaemic retina with persistent vitreous attachment. Within the posterior pole, vitreous and pre‐retinal haemorrhage likely results from tractional forces and shear stress exerted on the area of neovascularisation bound to the retinal circulation.^74^

Prior to the emergence of OCT‐A, evaluation of early retinal ischaemia in DR was limited to ophthalmologists with access to fluorescein angiography. Current generation OCT‐A enables rapid, non‐invasive visualisation of areas of reduced capillary perfusion, enlargement and distortion of the foveal avascular zone (FAZ) and pruning of the arteriolar branches (Figure 4B). OCT‐A has also shown capillary impairment associated with intra‐retinal microvascular abnormalities, cotton wool spots or anomalous vascular loops and thus may be useful in staging disease severity.^65^ Areas of capillary non‐perfusion appear to be better delineated on OCT‐A than with fluorescein angiography or clinical examination.^61^, ^73^, ^77^ Capillary closure may occur in early stages of DR, worsening as DR progresses; thus measurements such as FAZ size could potentially represent biomarkers for DR progression.^61^, ^77^ Krawitz et al.^78^ found a strong correlation between acircularity of the FAZ and the presence of DR.

### Key points

- DR is characterised by a diverse series of specific ophthalmoscopic signs. The appearance of these signs using OCT and OCT‐A has been well‐described in the literature; however, the specific application of OCT in eyes without DR on clinical examination has not been fully determined.
- OCT is an indispensable adjunct for the detection and management of all sight‐threatening DMO.
- *Limitations*: OCT and OCT‐A for the assessment of intra‐retinal microvascular abnormalities and neovascularisation is presently limited by field of view. The application of OCT and OCT‐A to DR assessment in treatment‐naïve patients is evolving and may, in the future, include routine evaluation of the posterior vitreous interface and FAZ.

## Glaucoma

**Figure 6:**
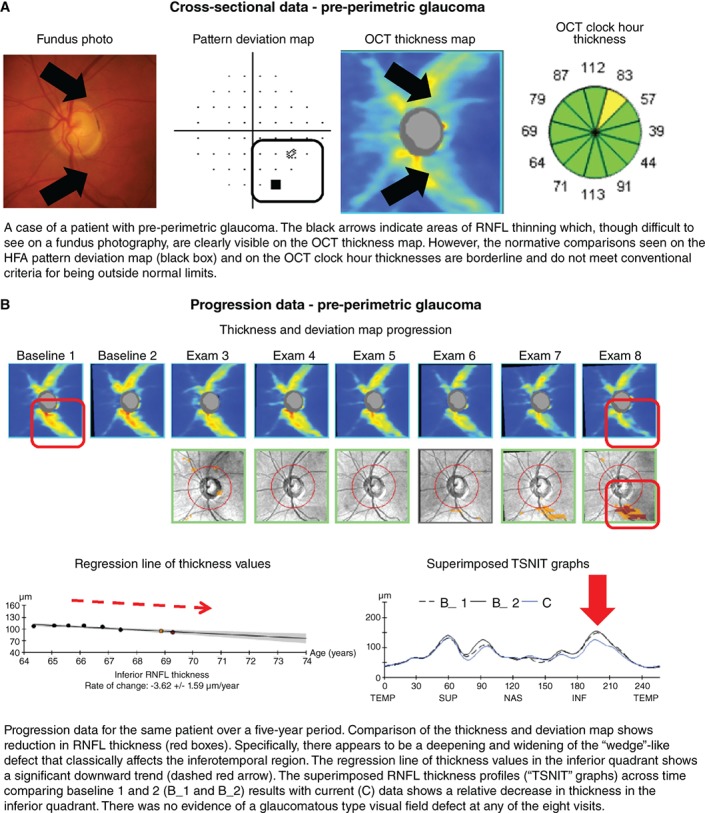
A: Cross‐sectional and B: progression data acquired using Cirrus HD‐OCT (optical coherence tomography) in a case of pre‐perimetric glaucoma. Abbreviations: as with Figures 1, 2, 3, 4, 5; HFA, Humphrey visual field analyser; RNFL, retinal nerve fibre layer; TSNIT, temporal superior nasal inferior temporal.

The definition of glaucoma has evolved considerably over the past few decades.^79^, ^80^, ^81^ Beginning in the early twentieth century with a simple definition of observable characteristic optic nerve head and retinal nerve fibre layer changes with corresponding visual field loss in the presence of elevated intraocular pressure, the definition has since developed into one that is multifaceted and complex.^80^ Recent definitions have revisited the notion that structural deficits characteristic for glaucoma must be present,^82^, ^83^, ^84^, ^85^ even in the absence of other clinical features such as visual field deficits.^86^, ^87^ In other words, there has been a paradigm shift toward definitions with a structural, rather than functional, emphasis, especially in light of structure–function discordance and the stage of disease known as ‘pre‐perimetric glaucoma’ (Figure 6A).^88^, ^89^, ^90^

### Using OCT signs in glaucoma for clinical management

There are now several useful markers for identifying glaucomatous change in OCT results. Most commonly, optic nerve head parameters (such as the disc and rim area, cup‐to‐disc ratio, or cup volume), retinal nerve fibre layer thickness values or ganglion cell layer thickness at the macula are compared against normative data using algorithms inbuilt to each device. The clinician then uses those findings to guide their clinical decision making. Asymmetry in structural findings on OCT is another key marker of early stage disease. Characteristic structural defects in glaucoma include ‘wedge’‐like and ‘arcuate’‐like retinal nerve fibre layer defects that mimic and correspond with visual field loss, especially those in ‘classically’ affected anatomical areas (Figure 6A, B).^91^, ^92^, ^93^, ^94^, ^95^, ^96^ These characteristic OCT structural changes are assumed to be surrogate measurements of the anatomical changes that underpin glaucoma. However, this has been challenged by studies examining the pathophysiology of glaucoma describing cellular changes of the retinal ganglion cells which may not be adequately captured by conventional OCT instruments which measure the optical properties to infer thickness values.^97^, ^98^, ^99^

One of the key advantages of OCT is the quantification of structural information, which is invaluable for longitudinal analysis.^91^, ^100^, ^101^ As glaucoma is typically progressive, and since progression rate is critical for treatment titration,^102^, ^103^ this information is useful for guiding management decisions (Figure 6B). Longitudinal analysis is also dependent upon the fidelity of the measurement: in this case, this includes correct registration of the scan to account for subtle differences in optic nerve head position between visits and scans.^104^ With advancing disease, this registration may become difficult due to the loss of neuronal tissue and the neuroretinal rim at the optic nerve head.^105^

### Non‐traditional indicators of glaucoma using OCT

**Figure 7:**
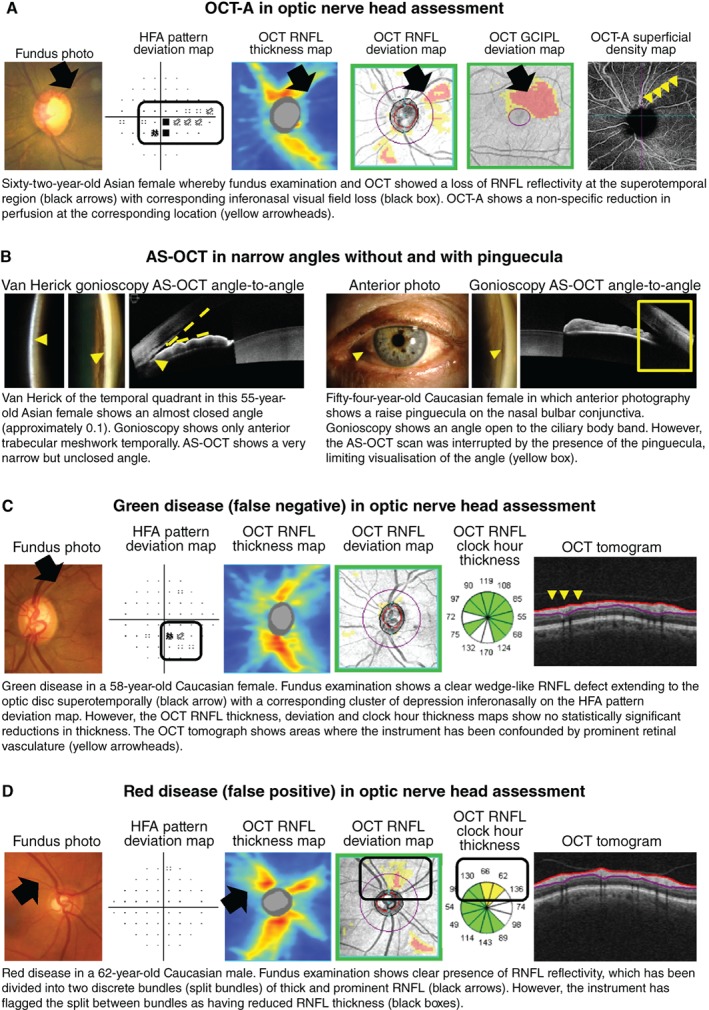
A: Optical coherence tomography angiography (OCT‐A) for evaluating anomalies in vascular perfusion, and B: anterior segment (AS) OCT for adjunctive assessment of the angle. C, D: Examples of glaucoma‐related green and red disease (false negatives and false positives), respectively. Abbreviations: as with Figures 1, 2, 3, 4, 5, 6; GCIPL, ganglion cell inner plexiform layer.

Ocular perfusion (both at the optic disc and peripapillary retina) may be visualised and measured using OCT‐A. The technology has recently garnered significant interest,^106^, ^107^ because alteration in vascular perfusion has also been suggested as a potential pathway to glaucoma.^108^, ^109^, ^110^ However, the technique has met with limited success due to the significant overlap between normal and glaucoma subjects and the non‐specificity of disease signs (Figure 7A).^111^, ^112^, ^113^

Another opportunity presented by OCT is that it makes some clinically ‘invisible’ structures visible. Two examples are the delineation of the anatomically true disc margin^114^, ^115^ and the lamina cribrosa using a volume scan approach.^116^, ^117^ Given the importance of optic disc size on describing optic nerve head parameters relevant for glaucoma,^118^ accurate delineation of the disc margin is critical to clinical decision making.^119^, ^120^, ^121^ The lamina cribrosa has also been hypothesised to play a role in the pathogenesis of the disease.^122^ New, high‐resolution OCT has been able to visualise the lamina cribrosa, describing parameters such as pore size and deformity.^123^ The significance of both of these parameters is still not fully known, but provides an exciting area for future study.

Aside from the optic nerve head, OCT is also used for assessment of the anterior segment.^124^, ^125^ Anterior segment OCT has shown promise in identifying abnormalities in key features within the anterior chamber angle by highlighting eyes that can develop angle closure and glaucoma, particularly in high‐risk populations (Figure 7B).^126^, ^127^, ^128^ However, there remains a significant number of limitations in anterior segment OCT, including resolution, feature identification, obstructing structures and static viewing, for example, in comparison to methods such as ultrasound biomicroscopy and gonioscopy.^129^, ^130^, ^131^, ^132^ Therefore, similar to examination of the posterior segment, anterior segment OCT remains an adjunct to gonioscopy, the current gold standard of anterior chamber angle assessment.^133^

OCT therefore appears to be an attractive option for visualising structural changes in a rapid manner, reinforced by recent studies that have provided precise visualisation of structural loss preceding significant visual field defects.^134^, ^135^, ^136^ It is not surprising that OCT has become indispensible for clinical glaucoma assessment, especially when its speed, precision and relative objectivity contrasts so heavily with the arduous, subjective and variable task of automated perimetry.^137^, ^138^

### Limitations of OCT imaging in glaucoma

**Figure 8:**
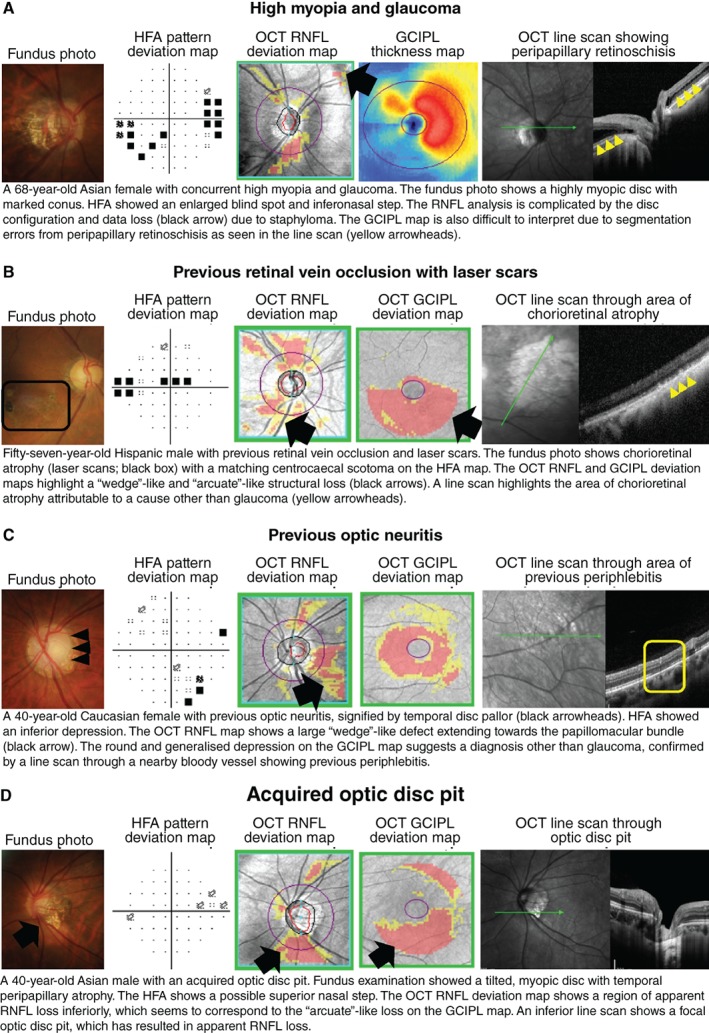
Case images illustrating the usefulness of optical coherence tomography (OCT) for differential diagnoses relating to glaucoma, including A: high myopia, B: retinal vascular occlusions, C: optic neuritis, and D: acquired optic disc pit. Abbreviations: as with Figures 1, 2, 3, 4, 5, 6, 7.

Given these advantages, is there still a place for tests aside from OCT for assessing glaucomatous damage? As mentioned already, there are a number of key limitations with using OCT imaging in glaucoma. OCT artefacts in glaucoma assessment^139^ may contribute to the manifestation of red‐green disease (for example, Figure 7C, D),^140^, ^141^ as determination of the statistical significance of structural loss is contingent upon the underlying normative database.^142^, ^143^ This is particularly relevant in cases of patient demographics that are not well‐represented by the normative database (for example, high myopia; Figure 8A). There has been interest in developing normative data for these demographics but there remain challenges associated with the determination of normality in such patients.^144^

Another critical limitation of OCT is the instrument measurement floor in advanced disease.^145^, ^146^, ^147^ Remaining non‐neuronal cells or retinal vasculature produce a resultant thickness measurement that is not indicative of actual neuronal change relevant in glaucoma.^105^, ^148^ In such cases, visual field results may be more reliable and informative. This has given rise to the idea of different utility of OCT and visual fields in different stages of glaucoma.^149^ More recent studies have clarified the discordance in early glaucoma by using different perimetric stimuli.^150^, ^151^, ^152^, ^153^ However, the issue with current OCT results providing only limited dimensional information of optic nerve head structure remains. This is an inherent limitation of an optical impression of ocular structure that does not truly represent the underlying anatomy.

Unlike in AMD and DR, OCT imaging of treatment‐naïve eyes with glaucoma does not necessarily yield ‘classic’ signs, and instead displays significant overlap with other disorders affecting the optic nerve head, such as retinal vascular occlusions and ischaemic optic neuropathies (Figure 8B–D).^154^, ^155^ This further reinforces the idea of glaucoma as a disease of statistical abnormality (for example, statistically depressed visual field or OCT results).^145^ As alluded to above, other optic nerve head appearances or variations may mimic glaucomatous damage, and provide a false impression of pathological changes that may be better assessed using alternative clinical techniques (for example, tilted disc syndrome or myopic optic discs).^156^, ^157^ OCT in isolation, therefore, does not clearly provide a conclusive diagnosis of glaucoma.

### Key points

- There exist a number of useful OCT markers for diagnosing glaucoma, particularly in early stages, including structural asymmetry, characteristic ‘wedge’‐like and ‘arcuate’‐like defects, and change over time.
- The measurement of optic disc and peripapillary perfusion using OCT‐A, structural OCT imaging of the lamina cribrosa, and anterior segment OCT applied for visualisation of the anterior chamber angle may be helpful in separating eyes with glaucoma from suspects.
- *Limitations*: Glaucoma is a disease of statistical abnormality and thus, OCT in isolation cannot provide a conclusive diagnosis of glaucoma. The usefulness of OCT in advanced glaucoma is limited by the instrument measurement floor.

## Future directions

Like retinal photography before it, OCT is now set to become a mainstay of primary eye care in developed nations. Already we are seeing the relegation of scan acquisition to non‐specialised personnel and injudicious use of the technology for disease screening rather than diagnostic purposes. Described thus far, there may be an immediate practical benefit to the identification of high‐risk AMD cases if the specific OCT signs are carefully predetermined and identified. Similarly, the diagnosis of early DMO using OCT may provide a stimulus for improved glycaemic control, thus imparting other benefits with regard to other systemic complications of diabetes, as well as prompting early referral to an ophthalmologist for closer monitoring. In glaucoma, an ideal screening test has remained elusive, but OCT purportedly offers several advantages over previously suggested screening methods such as intraocular pressure, cup‐disc ratio and screening visual field paradigms.^158^, ^159^, ^160^ But results for the use of OCT as a screening device in glaucoma have been mixed.^161^, ^162^, ^163^, ^164^, ^165^, ^166^ As described above, there are no clear diagnostic ‘signs’ of glaucoma identifiable using OCT. Screening protocols are also contingent upon normative comparisons, which in turn are dependent upon a reference standard.

A corollary question is whether screening with OCT is analogous to lead‐time bias in diseases such as cancer, that is, is there a benefit in early case identification where the outcome may actually be the same?^167^, ^168^ Major clinical trials have highlighted the benefits of treatment in early glaucoma,^169^, ^170^, ^171^, ^172^ but these have not necessarily used or reported imaging technologies such as OCT in the case identification process. Several recent studies^88^, ^89^ have highlighted the benefits of treating pre‐perimetric glaucoma, but these again have focused on traditional methods of structural examination, for example, fundus photography. Integration of OCT into such clinical trials would be further informative, specifically for determining its suitability as a potential endpoint. Thus, while OCT is a powerful technique for visualising structures in ocular disease and shows great promise as a potential screening tool in primary care settings,^173^ there are a significant number of limitations that relegate it to an adjunctive tool. From a pool of 10,000 patients and assuming a glaucoma prevalence of 3.4 per cent, 340 will have glaucoma. Assuming 80 per cent sensitivity and 95 per cent specificity (the average across four studies),^161^, ^162^, ^163^, ^164^ 272 cases and 9,177 normal patients will be correctly identified. Yet, 483 normal patients will be falsely identified as glaucoma and 68 cases with glaucoma will be missed. Hence, screening for ocular disease using OCT alone is not recommended and the interpretation of OCT needs to be performed in conjunction with other clinical data (for example, in glaucoma, stereoscopic optic nerve head, fundus photography, intraocular pressure, visual fields, gonioscopy, pachymetry et cetera).

### OCT as a diagnostic rather than a screening tool

**Figure 9:**
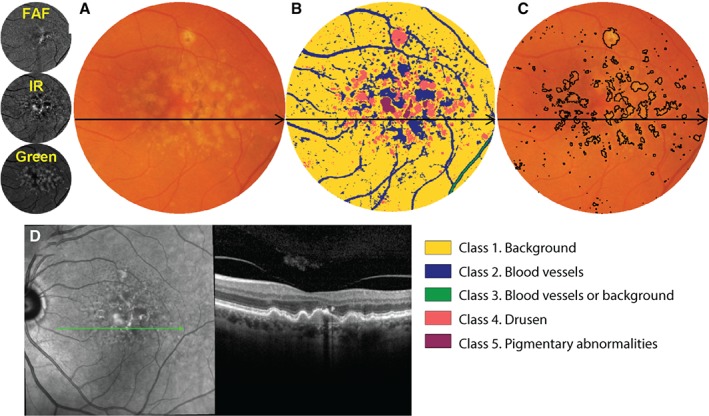
Demonstrative example of a semi‐automated strategy for integrating results of multiple imaging modalities. In this instance, a computational approach (unsupervised clustering) has been applied to the combination of infrared, autofluorescence and green scanning laser ophthalmoscopy findings to classify lesions in age‐related macular degeneration (AMD).^174^ A: Colour fundus photograph, cropped and masked to include the macular area only and B: corresponding classified image using this ‘pattern recognition approach’. Each distinct colour corresponds to a specific anatomical structure, as indicated by the figure legend. C: The class corresponding to drusen may then be outlined as a reflection of overall drusen load, which is an established risk factor for progression in AMD. D: Although colour fundus photography is the current standard of care in AMD grading, an additional optical coherence tomography (OCT) line scan has been provided to illustrate the drusen and pigmentary abnormalities. The same approach may also be applied for change analysis, that is for the surveillance of drusen load over time.^174^ FAF, fundus autofluorescence; IR, infrared.

The sensitivity for neovascular AMD and DMO may be higher than in glaucoma but is similarly not infallible. When using OCT alone and without knowing what to look for, neovascular AMD may be difficult to distinguish from other presentations, such as subretinal fibrosis, acquired vitelliform lesions or sub‐RPE haemorrhage, such as arising from polypoidal choroidal vasculopathy. Macular oedema and retinal nerve fibre layer loss can also arise from several causes, not just DR or glaucoma. A multimodal, evidence‐based imaging approach is therefore more informative and minimises the likelihood of diagnostic error. Although time‐consuming, numerous automated and semi‐automated strategies are evolving to make this process easier and more accessible in routine clinical practice (Figure 9).^174^

Machine learning in conjunction with OCT and other clinical data have been posited as a potential means to address challenges in chronic disease eye care, such as increasing prevalence, under‐diagnosis and workforce consumption.^175^, ^176^, ^177^ Intriguingly, machine and deep learning can provide a method for the interpretation of OCT by rapidly analysing aspects of the scan beyond that typically examined by human clinicians, such as volumetric A‐scan data. Deep learning algorithms are already able to automatically identify intra‐retinal and subretinal fluid.^178^ Given the importance of these signs, we are likely to see automated detection techniques incorporated to enhance the functionality of existing OCT devices in the near future. Automatic interpretation of OCT results and their combination with other aspects of the clinical examination provides another potential test in chronic eye disease.^179^, ^180^ Could screening with OCT alongside at least fundus photography therefore provide a means to address the problem of under‐diagnosis while simultaneously reducing the manpower required to detect disease? One of the biggest factors contributing to resistance to this technology is the ‘black box’ nature of the algorithm: the method by which the machine ‘learns’ and ‘interprets’ the results to come up with the confidence of diagnosis is unknown.^181^, ^182^ While human assessors are able to provide feedback regarding the decision‐making process, artificial intelligence systems may still be treated with some level of suspicion due to the number of hidden layers that underpin their decision‐making matrix. For the time being, clinicians using OCT should be able to apply the instrument judiciously and to translate their clinical observations into an appropriate management plan for the benefit of their patients.^183^ A number of resources are available to facilitate this translation, including printed education materials (https://centreforeyehealth.com.au/chairside‐references/).

## Conclusion

It could be argued that we are at a cross‐roads regarding the use of OCT in clinical practice. It may be applied *en masse*, that is for the indiscriminate, opportunistic screening of all patients presenting for an eye examination, or alternatively in a more judicious, targeted manner as a supplementary, diagnostic procedure. The discourse above illustrates the myriad of signs and considerations in just three ocular conditions, AMD, DR and glaucoma, let alone the innumerable number of others that may present during routine primary care. Ultimately, our role as clinicians is to prevent or minimise vision‐related disability and improve vision‐related quality of life. The time taken to accurately interpret OCT, particularly the most recent iteration, OCT‐A, is not insignificant. Consequently, the time, costs, complexity and risks involved in applying OCT should be weighed carefully against the benefits. The decision of when to use OCT should and must be evidence‐based.
